# Case Report: A novel compound heterozygosity of the *EVC*2 gene identified in a Chinese pedigree with congenital heart defect

**DOI:** 10.3389/fped.2025.1352571

**Published:** 2025-07-14

**Authors:** Xiayuan Xu, Chengcheng Gao, Keqin Jin, Liping Zhang, Yanfen Yang, Jun Zhang, Yun Ye, Shuangshuang Shen

**Affiliations:** ^1^ Department of Genetic Laboratory, Jinhua Maternity and Child Health Care Hospital, Jinhua, China; ^2^ Jinhua’s Key Laboratory of Birth Defects Prevention and Treatment, Jinhua Maternity and Child Health Care Hospital, Jinhua, China; ^3^ Key Laboratory of Digital Technology in Medical Diagnostics of Zhejiang Province, Dian Diagnostics Group Co., Ltd., Hangzhou, China; ^4^ Department of Ultrasonography, Jinhua Maternity and Child Health Care Hospital, Jinhua, China; ^5^ Department of Prenatal Diagnostic, Jinhua Maternity and Child Health Care Hospital, Jinhua, China

**Keywords:** congenital heart defect, fetus, neonate, *EVC2*, Ellis–van Creveld syndrome, case report

## Abstract

**Background:**

Congenital heart defects (CHDs) represent the leading cause of neonatal mortality among congenital abnormalities. Genetic factors, such as *EVC*2 gene mutations and other genetic alterations, constitute a major cause of CHD. Thus, determining the genetic etiology of fetal CHDs is crucial for optimizing pregnancy management and informing future reproductive decisions.

**Case presentation:**

Here, we describe a male fetus with complex CHD who was diagnosed at 25 weeks of gestation, delivered at full term, and died prematurely within a month due to heart failure. The cardiac abnormalities observed included an atrial septal defect developing from a patent foramen ovale, mitral valve regurgitation, dilated right ventricle and left atrium, aortic stenosis, and aortic arch dysplasia. Novel compound heterozygosity of the *EVC*2 gene, including a non-sense mutation (p.W828Ter) and two cis missense mutations (p.E87G and p.S217C), was identified by prenatal trio-whole-exome sequencing of amniotic fluid, followed by validation using Sanger sequencing. This novel *EVC*2 genotype was supposed to potentially affect fetal cardiac development, given the variable clinical heterogeneity of the *EVC*2 mutation-associated phenotype. This case represents the first identification of the *EVC*2 p.E87G and p.S217C, and the isolated CHD without visible skeletal dysplasia is an important feature of our case.

**Conclusions:**

Our study expands the genotypic and phenotypic spectra of *the EVC*2 gene. We recommend including the *EVC*2 gene in preconception carrier screening and prenatal diagnosis for CHDs.

## 1 Introduction

Congenital heart defects (CHDs) represent a large and rapidly emerging health challenge for neonates worldwide (1). Genetic alterations, including aneuploidy, copy number variation, and inherited sequence variation, can lead to the development of CHD (2, 3). It is essential to determine the cause of CHD in the proband based on the current medical progress as thoroughly as possible, which will help prevent mothers from giving birth to another child with CHD.

Ellis–van Creveld syndrome (EVCS) (MIM: #225500), also called chondroectodermal dysplasia, is a rare autosomal recessive disorder mainly caused by mutations in *EVC* or *EVC*2 genes and one of the most severe CHDs in neonates (4, 5). EVCS affects 1 in 60,000–200,000 neonates worldwide. Its characteristic features include short limbs and ribs, postaxial polydactyly, dysplastic nails and teeth, and congenital heart defects (6). Congenital heart defects, most commonly an atrial septal defect, are observed in 60% of affected individuals and contribute to the majority of deaths in EVCS infants (7, 8). Other cardiovascular malformations include persistent left superior vena cava (LSVC), unroofed coronary sinus, and anomalous pulmonary veins (9). The prenatal anomalies of EVCS could be identified as early as 18 weeks of pregnancy (10). However, the phenotypes of EVCS vary among individuals, ranging from lethal to mild clinical presentations. Formal evidence-based diagnostic criteria for EVCS have not been established, and the clinical diagnosis mainly relies on genetic sequencing (molecular diagnosis), clinical characteristics, and radiographic features. Thus, more research is needed to establish the association between *EVC*2 mutations and their phenotypes.

In the present study, we describe a male fetus diagnosed with complex CHD at 25 weeks of gestation. Biallelic *EVC*2 mutations (p.W828Ter, p.E87G, and p.S217C) were identified by prenatal trio-based whole-exome sequencing (trio-WES) of amniotic fluid.

## 2 Case presentation

### 2.1 Case description

**Figure 1:**
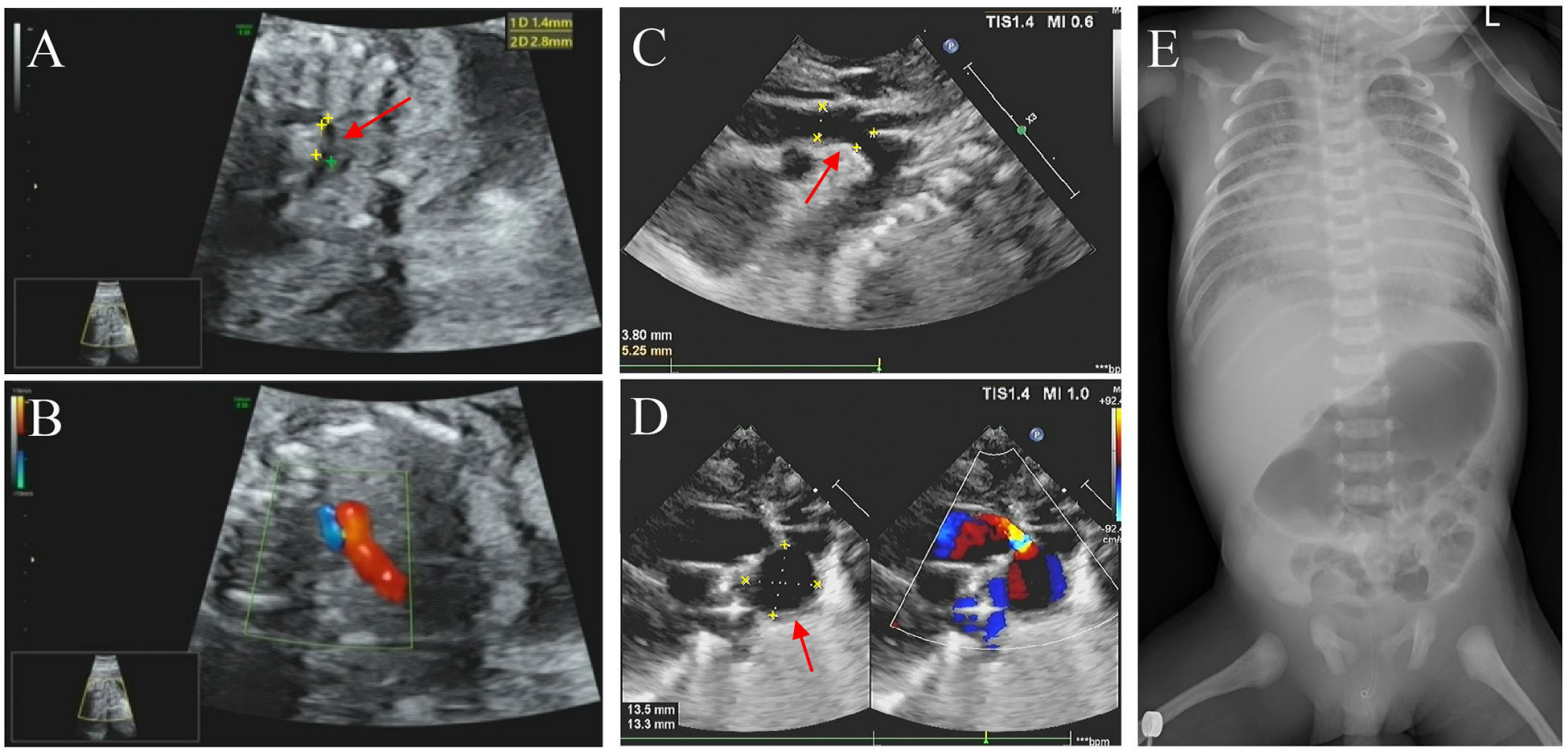
Imaging examination results. **(A,C)** Echocardiography performed during the fetal period (25 weeks pregnant) and neonatal period, respectively, showed the coarctation of the aortic arch (marked with an arrowhead). **(B)** The fetus exhibited pulmonary and aortic blood flow incongruity with an increased vessel ratio. **(D)** Neonatal imaging showing a patent ductus arteriosus aneurysm (marked with an arrowhead) and a bidirectional arterial shunt. **(E)** Digital x-ray imaging did n't show any obvious developmental abnormalities of the sternum, ribs, or spine.

In this family, the father is of Han Chinese descent, while the mother belongs to the Miao ethnic minority group in China. The parents denied a consanguineous marriage and have a healthy daughter. No family history of genetic disorders was reported. The proband was a male fetus, the second child of the 26-year-old mother (gravida 5, para 2). He was diagnosed with CHD *in utero* at 25 weeks of gestation. Fetal echocardiography revealed several cardiac abnormalities, with a normal cardiac rate (144 bpm); these abnormalities included aortic stenosis with coarctation of the aortic arch (from aortic root diameter measuring 2.3 mm to an isthmus of 1.7 mm), mitral valve regurgitation with reduced mitral valve flow, a patent foramen ovale (left-to-right shunt), an enlarged left atrium (left 13 mm × 13 mm vs. right 11 mm × 11 mm), and an enlarged right ventricle (right 14 mm × 11 mm vs. left 15 mm × 8 mm) (Figures 1A,B). The mother did not terminate the pregnancy despite the obstetrician informing her of the risks. At 39 weeks of gestation, the obstetrician reported no other abnormalities except the CHD.

The proband was delivered with normal growth parameters (birth weight = 3,130 g, length = 50 cm) by cesarean section at 40 weeks of gestation due to severe preeclampsia and then transferred to neonatology for respiratory distress. Imaging examinations did n't reveal any significant developmental anomalies of the sternum, ribs, or spine, but they did reveal more severe CHD symptoms, including an atrial septal defect (3.9 mm), a patent ductus arteriosus aneurysm (DAA, 14 mm × 13 mm aneurysm), aortic stenosis, aortic arch dysplasia (3.8 mm), moderate mitral regurgitation, an enlarged right ventricle (right 30 mm × 15 mm vs. left 27 mm × 16 mm), and an enlarged left atrium (left 26 mm × 23 mm vs. right 23 mm × 19 mm) with reduced left ventricular systolic function (Figures 1C–E). On the ninth day of life, the proband was transferred to the neonatal intensive care unit (NICU) due to heart failure (N-terminal BNP >5,000 pg/ml, troponin = 2.450 μg/L), respiratory failure, gastrointestinal bleeding, and cardiogenic shock accompanied by metabolic disorders (hypoglycemia, hyperlactatemia, and hyperkalemia). Life support measures were initiated, including ventilator-assisted ventilation, milrinone injection, alprostadil injection, and nutritional support therapy. Although medical treatment improved his metabolic acidosis and internal hemorrhage, the proband unfortunately died prematurely within a month due to heart failure.

**Figure 2:**
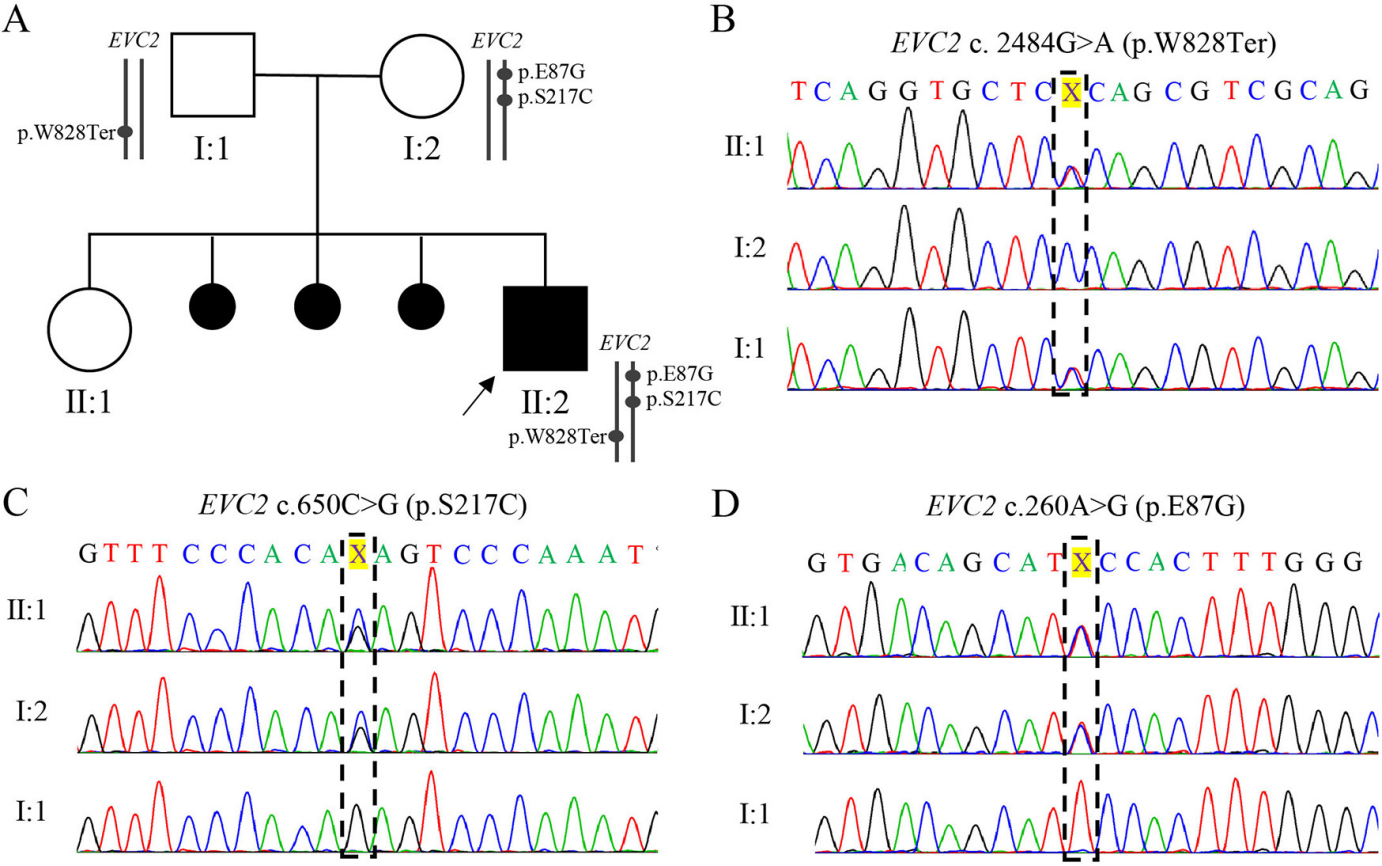
Family pedigree and Sanger sequencing validation of three *EVC*2 mutations. **(A)** The family pedigree. The cause of the last three miscarriages remains unknown due to the absence of preserved samples for further detection. **(B–D)** Chromas showing the mutation in the family. The position of *EVC*2 p.W828*, p.S217C, and p. E87G is marked with a black box.

Karyotyping and chromosomal microarray analysis (CMA) did n't reveal any abnormalities during pregnancy. However, prenatal trio-WES of amniotic fluid identified compound heterozygosity in *EVC*2 gene, consisting of a paternal non-sense mutation (NM_147127.5: c. 2484G>A, p.W828Ter) and two maternal missense mutations (c.260A>G, p.E87G; c.650C>G, p.S217C) (Table 1, Figure 2A). These three *EVC*2 mutations were verified by Sanger sequencing (Figures 2B–D). The *EVC*2 p.W828Ter mutation has been indexed as pathogenic or likely pathogenic for EVCS (ClinVar: #553315), while the other two *EVC*2 mutations have n't been previously reported or indexed in the database. The EVC2 protein contains a glycosylation site at amino acid position 220 and an EVC2-like domain spanning amino acids 237–660. None of the three detected mutations are located within the known EVC2 protein functional domains. The non-sense mutation, *EVC*2 p.W828Ter, is located in exon 14 and is predicted to lead to non-sense-mediated mRNA decay (PVS1 criterion met). According to GnomAD, the allele frequency (AF) for p.W828Ter is 0.0003013 in the East Asian population (PM2_supporting criterion met), while the other two missense mutations have not been indexed (PM2_supporting). The non-sense mutation has been reported in trans with pathogenic variants (*EVC*2 p.R399X and c.871-2_894del) in two patients with EVCS (PM3 criterion met) (11, 12). Integrated analysis—including rare exome variant ensemble learner (REVEL), combined annotation dependent depletion (CADD), ClinPred, and Computational_evidence_ratio—suggests that p.E87G is more likely to be tolerable, whereas most pathogenicity predictions for p.S217C are detrimental (Table 1) (meeting the PP3 criterion). Thus, *EVC*2 p.W828Ter is classified as pathogenic (PVS1 + PM2_supporting + PM3), while *EVC*2 p.S217C (PM2_supporting + PM3 + PP3) and p.E87G (PM2_supporting + PM3) are classified as variants of uncertain significance (VUSs). Unfortunately, samples from the last three miscarriages were not preserved for further verification. Thus, it is supposed that the novel identified *EVC*2 genotype may have affected the cardiac development of the proband.

**Table 1:** Database information and pathogenicity predictions of *EVC*2 mutations.

Characteristic	Mutation #1	Mutation #2	Mutation #3
Chromosome position	chr4:5624281	chr4:5699343	chr4:5690940
Nucleotide changes (NM_147127.5)	c.2484G>A	c.260A>G	c.650C>G
Amino acid changes (NP_667338.3)	p.W828Ter	p.E87G	p.S217C
Source	Paternal (Het)	Maternal (Het)	Maternal (Het)
ClinVar	P/LP^a^	Not indexed	Not indexed
Allele frequency	0.0003013	Not indexed	Not indexed
East Asian^b^	(6/19914)		
Literature	Zhang et al. (11)	—	—
	Zhuang et al. (12)		
SIFT	—	Detrimental	Detrimental
Polyphen2	—	Benign	Detrimental
Clinpred^c^	—	0.1258	0.8982
REVEL^d^	—	0.232	0.341
Computational_evidence_ratio^e^	—	0.0769	0.6923
CADD_phred^g^	—	10.29	20.3
Mutation classifications^f^	P	VUS	VUS

VUS, variant of uncertain significance.

The reference genome version is GRCh37.

^a^ P and LP mean pathogenic and likely pathogenic, respectively.

^b^ Data were from the GnomAD database.

^c^ Larger than 0.5 is supposed to be detrimental.

^d^ Larger than 0.75 is supposed to be detrimental.

^e^ Larger than 2/3 is supposed to be detrimental.

^f^ The determination of mutation classification was based on ACMG guidelines.

^g^ Scores between 10 and 20 are commonly used thresholds in CADD analysis. Variants with scores exceeding 10 are typically ranked within the top 10% in terms of predicted pathogenicity, while those with scores above 20 fall within the top 1%.

### 2.2 Genetic testing methodology

Trio-WES was performed using peripheral blood samples from the parents and amniotic fluid from the fetus. Genomic DNA was extracted from parental blood samples using the DNeasy Blood & Tissue Kit (Qiagen, Valencia, CA, USA), while fetal DNA was extracted from the amniotic fluid. The sequencing libraries were prepared using the Agilent SureSelect Human All Exon v.6. After PCR amplification, the sequencing libraries were sequenced on the HiSeq X Ten platform (Illumina Inc., San Diego, CA, USA). For all three individuals, the mean sequencing depth exceeded 175×, with more than 99.4% of variants covered at a depth greater than 20×.

Adapter sequences and low-quality reads were filtered out. The clean reads were then aligned to the human reference genome (GRCh37/hg19) using the Burrows–Wheeler Aligner (BWA). Variant calling was performed using the Genome Analysis Toolkit (GATK), followed by variant annotation using ANNOVAR. Candidate mutations were filtered based on AF (<1%), clinical phenotype, inheritance patterns, and pathogenicity classification.

Sanger sequencing was conducted to validate the candidate variants. The primer sequences used for amplification are the following: for *EVC*2 c. 2484G>A: forward 5′-TGCAGCAGGAGTGTTAGGTG-3′ and reverse 5′- CCTGCAGAACTCAGCCATGA-3′; for *EVC*2: c.260A>G, forward 5′-ACTGTGCACTAACGCTTCGG-3′ and reverse 5′-TTCACACTTCTGGATGAAAGTGC-3′; for *EVC*2: c.650C>G, forward 5′-ACATGCCTGACCCAGAACAC-3′ and reverse 5′-CGTGCTACCCTCCTTCTTCC-3′. Sequencing was performed using an ABI 3130 Genetic Analyzer (Applied Biosystems, CA, USA).

## 3 Discussion

A rising number of disease-causing genes and mutations have been identified in patients with CHD (13). In our CHD case, trio-WES identified novel biallelic mutations in the *EVC*2 gene. The *EVC*2 p.W828Ter mutation was reported as a pathogenic variant in two patients with EVCS (11). The other two missense variants, p.E87G and p.S217C, have n't been identified previously.

Notably, our male proband did n't exhibit visible skeletal dysplasia, which differs from the classic features of EVCS. This may be attributable to the high degree of variability in clinical expressivity. In a Pakistani family, three EVCS siblings carried a homozygous deletion mutation: *EVC* p.Leu244_Ser253delinsPro (14). Of these, two affected males only exhibited polydactyly, along with nail and dental abnormalities, while their sister presented with more severe symptoms, including short stature and CHD. Further study is warranted to determine whether there are differences in disease severity between female and male patients with EVCS. In another family, both compound heterozygous genotypes (p.Arg662Ter and p.Arg663Pro, p.Arg662Ter and c.1316-7A>G) were identified in individuals with EVCS (15). However, the individual carrying a compound heterozygosity genotype, p.Arg663Pro and c.1316-7A>G, only exhibited mild mitral regurgitation, indicating that some hypomorphic mutations could still produce a sufficient amount of functional EVC2 protein. Thus, it is hypothesized that the combination of *EVC*2 p.W828Ter, p.E87G, and p.S217C identified in our proband may induce CHD but have little effect on fetal skeletal development.

The EVC and EVC2 proteins constitute the EVC–EVC2 complex, located within the primary cilium. This complex functions by transducing extracellular signals to the nucleus via the Hedgehog (HH) signaling pathway (16). The pathogenesis of EVCS phenotypes caused by *EVC*2 mutations has been partially elucidated. *EVC2* loss-of-function results in delayed ameloblast differentiation by disrupting HH signaling in the dental epithelium, clarifying the pathogenesis of enamel hypoplasia in patients with EVCS (17). Apart from the HH signaling pathway, *EVC*2 mutations could induce dwarfism by elevating fibroblast growth factor (FGF) signaling (18). However, the pathogenesis of other phenotypes, especially the pathogenic mechanism of CHD, needs further research for clarification.

CHD related to *EVC*2 or *EVC* mutations can also be repaired surgically. Delayed surgery for CHD among children (1.3 vs. 50.1 months) was associated with fewer postoperative complications and a higher survival rate (19). Respiratory morbidity was identified as the main postoperative complication, while respiratory failure was the leading cause of mortality (20). However, our proband was born with respiratory distress and rapidly progressed to respiratory failure, accompanied by heart failure and cardiogenic shock, within 9 days. Consequently, surgical intervention was no longer appropriate. Thus, it is crucial to establish a clinical management process for infants with CHD carrying *EVC*2 mutations to get through early life until they are suitable for surgery.

Here, we report on the entire life course of a CHD fetus carrying compound heterozygous mutations in the *EVC*2 gene. The two novel *EVC*2 missense variants (p.E87G and p.S217C) were identified for the first time. It is hypothesized that this novel *EVC*2 genotype might affect heart development but have little effect on skeletal development. Our study provides new insights into prenatal diagnosis and genetic counseling for patients with CHD carrying *EVC*2 mutations. Meanwhile, we suggest establishing a clinical management process for CHD neonates carrying *EVC*2 mutations to prevent premature death.

## Data Availability

The data that support the findings of this study are available from the corresponding author upon reasonable request.
